# Effects of multiple acupuncture therapies on cognitive function and quality of life in stroke patients: a systematic review and network meta-analysis

**DOI:** 10.3389/fneur.2026.1764104

**Published:** 2026-03-18

**Authors:** Zixin Teng, Zhi Gao, Jingwei Zhu, Ling Zou, Xin Fu, Haoran Chu, Peiyang Sun

**Affiliations:** 1 First Clinical Medical College, Anhui University of Chinese Medicine, Hefei, Anhui, China; 2 Medical Affairs Department, The Second Affiliated Hospital of Anhui University of Chinese Medicine, Hefei, Anhui, China; 3 Guoyitang Clinic, The Second Affiliated Hospital of Anhui University of Chinese Medicine, Hefei, Anhui, China; 4 Encephalopathy Department, The Second Affiliated Hospital of Anhui University of Chinese Medicine, Hefei, Anhui, China

**Keywords:** acupuncture, cognitive function, network meta-analysis, quality of life, stroke

## Abstract

**Objective:**

To evaluate the comparative effectiveness of different acupuncture therapies in stroke rehabilitation using network meta-analysis.

**Methods:**

We systematically searched major databases for RCTs (2016–2025) comparing six acupuncture interventions. Frequentist network meta-analysis was conducted in Stata 17.0, with interventions ranked by SUCRA values. Primary outcomes included NIHSS, BI, mRS, MoCA, and MMSE scores.

**Results:**

Analysis of 120 RCTs (*n* = 15,848) demonstrated distinct efficacy profiles: EA ranked highest for neurological recovery (NIHSS SUCRA 95.7%) and BI improvement (74.8%); WNM was optimal for disability reduction (mRS SUCRA 100%) and comprehensive cognition (MoCA 94.9%); while SA excelled in MMSE improvement (86.1%).

**Conclusion:**

Distinct acupuncture modalities exhibit unique advantages in the context of stroke rehabilitation. EA ranked first in the restoration of neurological function, whereas WNM and SA were identified as the optimal interventions for global disability and cognitive impairment, respectively. Given that the included studies originated exclusively from China, the external validity of the present findings in other populations warrants further verification.

**Systematic review registration:**

https://www.crd.york.ac.uk/PROSPERO, CRD42025112036.

## 1 Introduction

As an acute cerebrovascular pathological event, stroke arises from the sudden occlusion or rupture of cerebral vessels, leading to subsequent brain tissue injury. The disease is broadly differentiated into ischemic and hemorrhagic categories, with current epidemiological evidence establishing that ischemic strokes constitute roughly 80% of all documented cases (1). Stroke-induced acute vascular events initiate a complex pathological process centered around an energy crisis, which cascades into excitotoxicity, calcium overload, and diffuse neuroinflammation (2), ultimately resulting in selective neuronal death or regional infarction (3). This process disrupts the functional integrity of brain networks and often leads to various sequelae such as motor, sensory, and cognitive impairments, significantly reducing patients’ quality of life. Epidemiological studies establish stroke as the second leading global cause of mortality and a predominant factor in disability-related outcomes (4), imposing considerable burdens on social systems and households. Consequently, preventive strategies and rehabilitative measures for stroke warrant coordinated international focus.

Conventional stroke management primarily focuses on etiology-specific recanalization, cerebral blood flow restoration, neuroprotective agents, and antiplatelet therapy during the acute phase. However, due to the narrow therapeutic window and considerable treatment risks (5), these approaches demonstrate limited efficacy, benefit only a small proportion of patients, and often fail to adequately improve neurological deficits or enhance quality of life (6). Modern medicine still lacks comprehensive strategies for long-term management of numerous dysfunctions during stroke recovery. Therefore, identifying and optimizing complementary and alternative therapies is particularly important.

As a cornerstone intervention within Traditional Chinese Medicine, acupuncture continues to demonstrate its expanding role in contemporary stroke rehabilitation protocols. Growing clinical evidence supports its efficacy in improving stroke-related cognitive, sensory, and motor impairments (7). Acupuncture exerts therapeutic effects through multiple pathways and targets, with different needling techniques demonstrating distinct effect profiles. Scalp acupuncture (SA) enhances regional brain activity and interhemispheric functional connectivity in ischemic stroke patients, improving sensory, motor, and language functions (8). It also elevates serum BDNF levels while reducing inflammatory markers to enhance cognitive recovery (9). Electroacupuncture (EA) combines needling with electrical stimulation at specific frequencies to sustainably activate neural pathways and ameliorate inflammatory responses and vascular and motor dysfunction (10). Peri-eye acupuncture (PEA) mitigates cerebral ischemia-induced neuronal damage by increasing cerebral blood flow, promoting angiogenesis, and stimulating neurogenesis (11). These multi-modal mechanisms target various aspects of neural repair, providing unique therapeutic value for stroke rehabilitation.

However, existing systematic reviews have largely concentrated on comparisons between single acupuncture modalities and conventional treatments, whereas direct comparisons among different acupuncture therapies are lacking. Consequently, clinicians lack high-level evidence to guide the selection of the optimal acupuncture regimen. Accordingly, the present study was designed to bridge this gap through a network meta-analysis. Strictly adhering to the PICO principle, we systematically compared various acupuncture modalities—including manual acupuncture (MA), SA, EA, PEA, warming needle moxibustion (WNM), and fire needling (FN)—to assess their relative efficacy in improving cognitive function and quality of life in stroke patients, with the aim of providing an evidence-based foundation for the clinical selection of the optimal acupuncture scheme.

## 2 Materials and methods

### 2.1 Protocol and registration

This study was prospectively registered with PROSPERO (registration number: CRD42025112036). The methodology conforms to the Preferred Reporting Items for Systematic Reviews and Meta-Analyses (PRISMA) guidelines and follows the PRISMA extension for network meta-analysis (12), thereby strengthening the methodological rigor and completeness of the review.

### 2.2 Search strategy

We systematically searched for RCTs investigating acupuncture interventions for stroke, published from 2015 through 2025. The retrieval spanned multiple databases, including PubMed, Web of Science, EMBASE, the Cochrane Central Register of Controlled Trials, CNKI, and Wanfang Data. The search methodology incorporated both controlled vocabulary and keywords related to the disease and interventions, focusing on the key themes of “Stroke,” “Acupuncture,” and “Randomized Controlled Trial.” The screening process was conducted without restrictions on language or publication location. The complete search approach for the English databases is outlined in Table 1 (taking the search strategy in PUBMED as an example). The complete search approach for the Chinese databases is outlined in Supplementary Table 1.

**Table 1:** Search strategy on PubMed.

Search strategy on PubMed	
#1	(((“Stroke”[Mesh]) OR ((((((((((((((((((((((((((((Strokes) OR (Cerebrovascular Accident)) OR (Cerebrovascular Accidents)) OR (Cerebral Stroke)) OR (Cerebral Strokes)) OR (Stroke, Cerebral)) OR (Strokes, Cerebral)) OR (Cerebrovascular Apoplexy)) OR (Apoplexy, Cerebrovascular)) OR (Vascular Accident, Brain)) OR (Brain Vascular Accident)) OR (Brain Vascular Accidents)) OR (Vascular Accidents, Brain)) OR (Cerebrovascular Stroke)) OR (Cerebrovascular Strokes)) OR (Stroke, Cerebrovascular)) OR (Strokes, Cerebrovascular)) OR (Apoplexy)) OR (CVA (Cerebrovascular Accident))) OR (CVAs (Cerebrovascular Accident))) OR (Stroke, Acute)) OR (Acute Stroke)) OR (Acute Strokes)) OR (Strokes, Acute)) OR (Cerebrovascular Accident, Acute)) OR (Acute Cerebrovascular Accident)) OR (Acute Cerebrovascular Accidents)) OR (Cerebrovascular Accidents, Acute)))
#2	((“Acupuncture”[Mesh]) OR (((((((Pharmacopuncture) OR (warm needle)) OR (fire needle)) OR (electroacupuncture)) OR (floating needle)) OR (abdominal acupuncture)) OR (laser acupuncture))))
#3	#1 AND #2
#4	((“Randomized Controlled Trials as Topic”[Mesh]) OR (“Randomized Controlled Trial” [Publication Type]))
#5	#3 AND #4

### 2.3 Inclusion and exclusion criteria

#### 2.3.1 Inclusion criteria

Established according to the PICOS principle:

(P) Population: Patients with stroke;

(I) Intervention: Acupuncture therapies including MA, SA, EA, WNM, PEA, ear acupuncture, FN, and thick needle acupuncture;

(C) Comparison: Control groups receiving conventional treatment (including symptomatic management and rehabilitation training), sham acupuncture, or other acupuncture interventions different from the experimental group;

(O) Outcomes: Assessment of cognitive function and quality of life in stroke patients using at least one of the following scales: National Institutes of Health Stroke Scale (NIHSS), Activities of Daily Living scale (ADL), modified Rankin Scale (mRS), Barthel Index, Montreal Cognitive Assessment (MoCA), or Mini-Mental State Examination (MMSE);

(S) Study design: RCTs.

#### 2.3.2 Exclusion criteria

(1) Reviews, animal studies, case reports, non-randomized controlled trials, retrospective studies, expert opinions, conference abstracts, and duplicate publications.
(2) Studies with inaccurate data or incomplete outcome measures where the original authors could not be contacted;
(3) Studies with fewer than 50 participants;
(4) Literature published before 2015;
(5) Studies with unclear or incomparable baseline characteristics.

### 2.4 Literature screening and data extraction

Literature screening was independently conducted by two reviewers using EndNote 20, followed by cross-verification. Data extraction was performed in Excel 2020, also by two independent reviewers. In both stages, any discrepancies were resolved through consensus or by consulting a third reviewer. The following data were extracted:

- publication details (title, first author, year);
- study characteristics (population, follow-up);
- participant information (sample size, age);
- intervention details (method, acupoint selection, frequency, needle retention, median follow-up);
- outcome measures (NIHSS, ADL, mRS, Barthel Index, MoCA, MMSE scores).

### 2.5 Literature quality assessment

The quality assessment procedure involved two independent reviewers utilizing the Cochrane Risk of Bias Tool (2011 version) (13). Following individual evaluations, a cross-validation process was implemented where assessment discrepancies were addressed through deliberation or by seeking arbitration from a third researcher.

### 2.6 Statistical analysis

For data synthesis, the standardized mean difference (SMD) with a 95% confidence interval (CI) was calculated as the pooled effect measure. We used Stata 17.0 to generate network diagrams, funnel plots, and cumulative ranking curves for the seven interventions, employing the mvmeta and network packages for the network meta-analysis. In the network diagram, nodes depict the distinct acupuncture therapies and control, and the connecting lines between them represent direct comparisons. The size of each node and the thickness of each line are proportional to the number of underlying studies. When closed loops were present in the network, both global and local inconsistency tests were conducted (14). A consistency model (*p* > 0.05) or inconsistency model (*p* < 0.05) was selected based on the test results. Pairwise comparison results were presented using league tables, while the ranking of interventions was expressed through the Surface Under the Cumulative Ranking Curve (SUCRA) (15). Funnel plots were generated to assess publication bias and small-study effects. To evaluate the robustness of the pooled results, sensitivity analyses were conducted. Specifically, studies with an “unclear” or “high” risk of bias in random sequence generation, as well as those with a “high” risk of bias in the blinding of participants and personnel, were excluded. The pooled effect sizes and treatment rankings were subsequently re-estimated to assess the impact of these methodological quality factors on the overall conclusions.

## 3 Results

### 3.1 Literature search results

**Figure 1:**
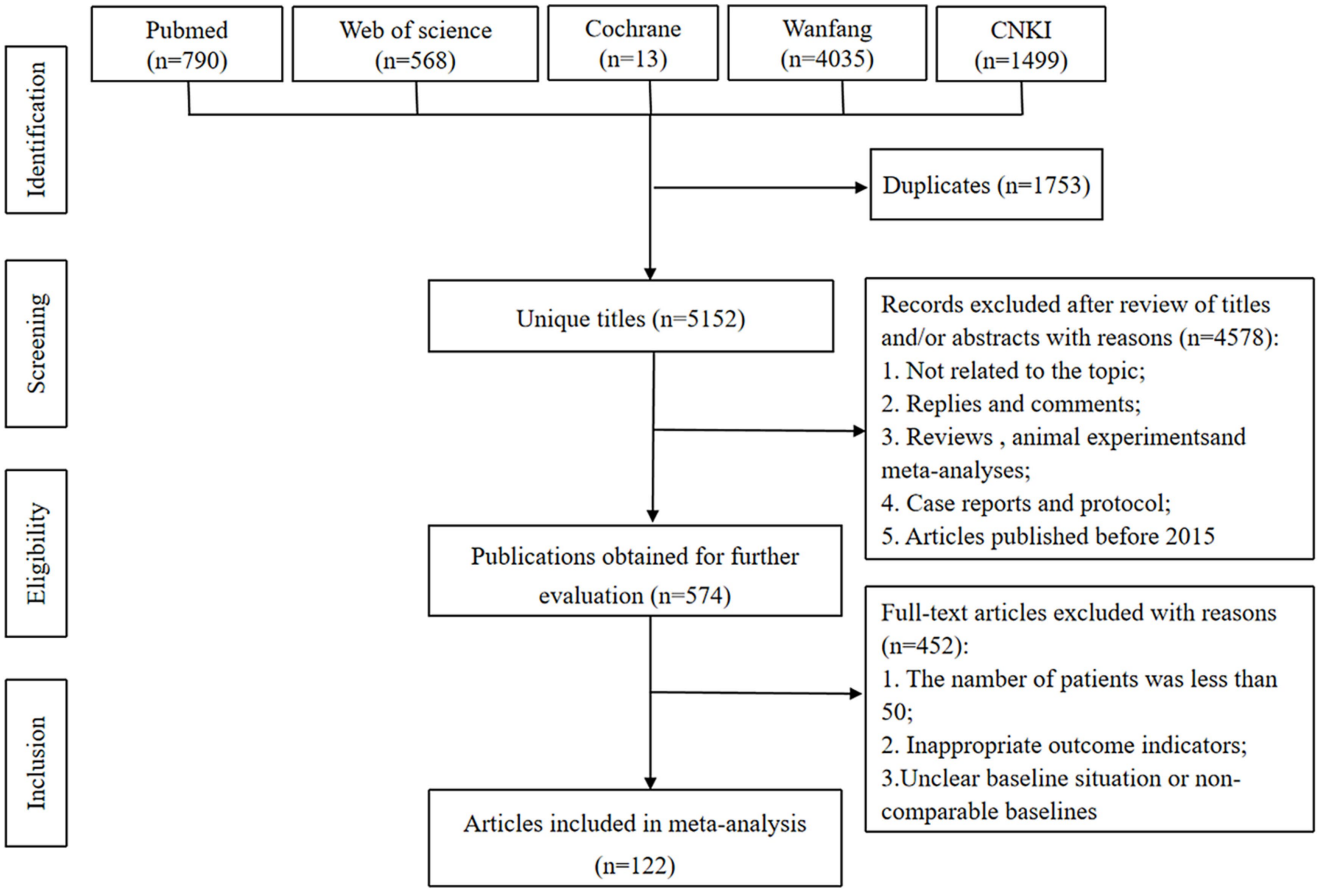
Literature screening flowchart.

The literature identification phase commenced with 6,905 potentially relevant citations. Deduplication eliminated 1,753 records, resulting in 5,152 unique publications undergoing preliminary screening based on titles and abstracts. This process led to the exclusion of 4,578 articles, with the remaining 574 proceeding to rigorous full-text examination. The final cohort comprised 120 studies that satisfied all predetermined eligibility criteria. Table 2 synthesizes the essential characteristics of the included investigations, and Figure 1 provides a schematic representation of the selection methodology.

**Table 2:** Basic information table of RCTs included studies.

Authors	Country	Study design	Patients (n)	Age(years)	Durational of disease(d)	Stroke Type	Intervention	Acupoint	Frequency (times/week)	Needle Retention (min)	Median follow-up (weeks)
			Acu/Con	Acu/Con	Acu/Con		Acu/Con				
Bao et al. (59)	China	prospective	60/60	58.42 ± 16.51/56.25 ± 10.40	-	Hemorrhagic	MA/UT	LI15, LI11, LI4, SJ5, LI10, ST36, GB34, GB30, ST34, ST35, ST40, SP6, ST41, KI3, BL62, LR3, GV20, EX-HN1, EX-HN5	7	20	3
Bi. (60)	China	prospective	51/51	64.2 ± 8.7/63.9 ± 8.9	1.4 ± 0.5/1.2 ± 0.4(momths)	Ischemic	MA/UT	GV20, EX-HN1, ST2, GB20, GV26, GB12, BL10, HT7, PC6, SP6, LR3, ST40	6	30	6
Cai et al. (61)	China	prospective	57/57	61.87 ± 5.19/61.57 ± 4.28	3.26 ± 0.48/3.19 ± 0.62	Mixed	MA/UT	EX-B2, GV16, GV1, GV24, GV20, PC6, GV26, GV23, EX-HN1	6	30	4
Cai et al. (62)	China	prospective	83/83	62.92 ± 9.71/63.09 ± 8.84	2.73 ± 1.30/2.85 ± 1.26	Ischemic	EA/UT	LR3, LI15, SP6, LI13, ST40, LI11, SP10, SJ5, GB34, PC6, ST36, LI4, LI10	7	30	4
Cao et al. (63)	China	prospective	54/54	56.3 ± 7.2/57.2 ± 7.3	29.1 ± 10.2/28.5 ± 9.71(h)	Ischemic	MA/UT	PC6, GV26, SP6, HT1, LU5, BL40	7	20	2
Zha et al. (64)	China	prospective	79/79	60 ± 7/59 ± 10	1.78 ± 0.56/1.87 ± 0.52	Ischemic	MA/UT	GV20, EX-HN1, LI15, LI11, LI10, ST36, SJ5, PC6, LI4, SP6, LR3, SP9, ST34	7	30	4
Chen. (65)	China	prospective	52/52	60.21 ± 8.18/60.49 ± 8.45	20.95 ± 5.01/22.16 ± 4.59	Mixed	MA/UT	GV20, LI11, PC6, LI4, GB34, ST36, ST40, SP6	7	30	1
Chen et al. (66)	China	prospective	50/50	65.66 ± 7.54/67.84 ± 7.11	13.38 ± 13.99/12.9 ± 15.84(h)	Ischemic	MA/UT	PC6, GV26, SP6, GV29	7	-	2
Chen and Guan (67)	China	prospective	55/55	48.2 ± 4.74/47.11 ± 5.61	-	Mixed	MA/UT	KI3, ST36, HT7, GV16	6	30	4
Chen et al. (68)	China	prospective	54/54	56 ± 7/57 ± 7	-	Ischemic	MA/UT	PC6, GV26, SP6, HT1, LU5, BL40	7	30	4
Chen et al. (69)	China	prospective	61/61	56.62 ± 5.68/55.21 ± 5.34	43.93 ± 8.87/42.34 ± 9.28	Mixed	FN/UT	Ashi point	3	-	4
Chen et al. (70)	China	prospective	56/56	48.73 ± 7.65/49.38 ± 8.59	-	Ischemic	MA/UT	SJ5, LI11, LU5, PC6, LI4, HT1	6	30	4
Chen and Lu (25)	China	prospective	53/53	59.31 ± 11.83/58.75 ± 12.12	23.35 ± 16.15/22.46 ± 15.62	Mixed	WNM/UT	GV20, EX-HN1, GB7, LI20, GV29, ST7, EX-HN5, GV26, ST4, GB20, SJ17, LI15, TE14, SI9, LI10, LI11, PC6, SJ5, LI4, SI1, SI3, GB30, GB31, BL54, ST36, GB34, SP6, BL57, KI1, BL60, KI3, ST44, LR3, CV4, CV6, RN8	5	30	12
(71)	China	prospective	70/70	54.8 ± 4.3/55.1 ± 4.7	-	Ischemic	PEA/UT	Eye area acupoints	5	30	2
Chu et al. (72)	China	prospective	60/60	71 ± 8/69 ± 8	8.2 ± 3.7/9.2 ± 3.3	Ischemic	MA/UT	LI15, LI11, LI10, SJ5, GB30, ST31, ST32, ST36, SP6	6	30	2
Dai. (73)	China	prospective	57/57	62.7 ± 8.6/62.9 ± 9.1	38.2 ± 10.4/62.9 ± 9.1	Mixed	EA/UT	GV20, EX-HN5, GB20, GV16, HT1, LI11, PC6, LI10, PC7, LI4, SI3, SP9, BL40, SP6, ST40, SP5, LR3, ST41, GB40	6	30	4
Dang et al. (74)	China	prospective	50/50	52.45 ± 4.06/52.28 ± 3.29	21.85 ± 2.27/22.09 ± 2.87	Mixed	MA/UT	PC6, ST36, SP6, SP10, HT1, LU5, BL40, GV26, EX-HN1, GV20, GV29	4	30	4
Dou et al. (75)	China	prospective	100/100	-	-	Mixed	MA/UT	GV20, LI11, LI15, ST36, GB34, GB30, BL40	3	50	9
Du et al. (76)	China	prospective	64/63	63.11 ± 6.98/63.89 ± 7.21	3.22 ± 1.37/3.4 ± 1.29	Ischemic	MA/UT	GV14, GV4, GV20, DU21, GV24, GV16	7	30	12
(77)	China	prospective	60/60	60.3 ± 5.1/60.2 ± 5.8	-	Ischemic	WNM/UT	BL13, BL15, BL23, GV24	7	30	6
Fang et al. (16)	China	prospective	54/55	61.35 ± 8.60/60.24 ± 8.26	51.59 ± 9.75/51.67 ± 9.59	Mixed	MA/UT	GV20, EX-HN1, GV24, SP6, KI3, GB39	6	30	8
Feng et al. (78)	China	prospective	65/69	65.2 ± 5.3/63.9 ± 6.8	-	Hemorrhagic	EA/UT	The area related to the head acupoints	5	25	8
Feng et al. (79)	China	prospective	65/69	64.1 ± 6.5/63.9 ± 6.8	-	Hemorrhagic	EA/UT	The area related to the head acupoints	5	25	6
Feng et al. (80)	China	prospective	50/50	48.32 ± 2.21/48.35 ± 2.20	-	Ischemic	EA/UT	HT1, PC3, LU5, PC6, LR9, HT3, SP10, SP6, ST36, LI4, SJ5, SI3, ST41, GB34, GB31, GB40	7	30	12
Feng et al. (81)	China	prospective	50/50	60.25 ± 1.18/60.23 ± 1.15	-	Ischemic	EA/UT	LI15, LI11, LI4, GB30, GB31, GB34, ST36	7	30	12
Feng et al. (82)	China	prospective	60/60	62.6 ± 6.2/63.1 ± 6.4	-	Ischemic	MA/UT	PC8, KI3, LI15, LI11, ST37	7	30	2
Ge. (83)	China	prospective	59/59	67.25 ± 8.57/68.11 ± 8.94	-	Ischemic	MA/UT	GV20, LI11, LI10, SJ5, LI4, ST36, GB34, SP6	5	30	4
Han et al. (17)	China	prospective	59/59	64 ± 9/66 ± 8	6.3 ± 2.6/6.7 ± 2.4(momths)	Ischemic	MA/UT	GV20, EX-HN1, CV12, ST26, CV10, CV6, CV4, KI19, KI17, ST25, ST40, ST24	5	30	8
Han et al. (84)	China	prospective	54/54	63.14 ± 4.58/63.23 ± 4.77	29.21 ± 4.18/29.11 ± 4.06	Mixed	MA/UT	LI15, LI4, LI11, SJ10, HT1, PC6, LU5, PC7, SP10, ST34, ST31, LR8, ST41, BL62	6	30	4
Han. (85)	China	prospective	64/64	60.5 ± 9.4/61.2 ± 10.5	80.3 ± 10.7/79.6 ± 11.3	Mixed	WNM/UT	ST36, CV4, LI11, LI15, SJ5	5	30	4
He et al. (86)	China	prospective	55/55	66.83 ± 3.16/66.13 ± 3.52	-	Ischemic	WNM/UT	HT1, PC6, LI11, SP6, BL40	5	30	4
Hu et al. (87)	China	prospective	76/74	65 ± 15/62 ± 12	27.2 ± 8.6/29.3 ± 3.2	Mixed	WNM/UT	LI11, LI10, LI4, LI15, TE14, SI9, SJ5, KI3, GB39, SP10, ST34, SP9, ST41, LR3	5	30	4
Hu and He (88)	China	prospective	54/54	46.8 ± 7.2/47.8 ± 6.3	21.9 ± 3.2/24.2 ± 3.1	Ischemic	MA/UT	GV26, SP6, PC6, LU5, BL40, HT1, LI10, ST36, SI9, LI11, LR3, ST40	7	-	4
Huang et al. (89)	China	prospective	60/60	61.87 ± 5.71/61.63 ± 5.92	4.36 ± 1.07/4.57 ± 1.16(momths)	Ischemic	MA/UT	GV20, PC6, SP6, LU5, HT1, BL40	3	30	8
Huang et al. (90)	China	prospective	62/62	-	-	Ischemic	MA/UT	GV20, EX-HN1, GV24, GB13	5	30	4
Jia et al. (91)	China	prospective	102/102	68.81 ± 5.53/68.74 ± 5.49	23.59 ± 4.68/23.47 ± 4.74	Mixed	MA/UT	LI15, LI11, PC3, LI4, PC8, SJ5, PC6, ST34, SP10, GB34, SP9, GB39, SP6, GB40, SP5	5	30	2
Jiang. (92)	China	prospective	58/58	58.47 ± 8.05/57.02 ± 7.88	-	Mixed	MA/UT	GV20, GV26, ST4, LI10, LI11, PC6, LI4, SJ5, TE14, SI1, ST36, GB30, GB34, GB31, SP10, BL57, SP9, SP6	5	30	4
Jin et al. (93)	China	prospective	60/60	57.48 ± 8.65/59.48 ± 8.45	18.22 ± 4.31/17.68 ± 4.24(weeks)	Mixed	MA/UT	CV12, CV10, CV6, CV4, ST24, ST26, GB39	3	30	8
Ke. (94)	China	prospective	81/81	58.6 ± 4.4/58.3 ± 4.2	2.9 ± 1.3/2.8 ± 1.5(h)	Ischemic	MA/UT	GV20, GB7, EX-HN1, PC6, LI4, ST36, GB34, SP6, ST40	7	40	4
(95)	China	prospective	62/62	65.17 ± 5.76/64.48 ± 5.62	7.28 ± 0.64/7.31 ± 0.58	Ischemic	MA/UT	PC6, GV26, GB20, GV29	7	30	4
Li et al. (96)	China	prospective	50/50	58.53 ± 8.41/58.95 ± 8.47	27.67 ± 6.04/27.35 ± 6.43	Mixed	WNM/UT	PC6, LU5, LI10, LI11, LI4, SP10, GB31, GB30, GB40, LR3, GB34	7	30	6
Li et al. (97)	China	prospective	130/130	62.82 ± 5.75/62.72 ± 5.84	5.94 ± 1.27/6.03 ± 1.21(h)	Ischemic	MA/UT	GV26, GV20, GV24, PC6, SJ4, SJ10, SP6, GB34, SP10	7	30	2
Li et al. (98)	China	prospective	55/55	54.37 ± 5.63/53.86 ± 3.28	2.73 ± 0.65/2.62 ± 0.31(momths)	Mixed	EA/UT	HT1, SJ5, LI4, LI11, LI10, PC7, GB30, LR8, ST36, SP6, LR3	7	30	6
Li et al. (99)	China	prospective	65/65	56.82 ± 8.01/56.94 ± 8.10	12.77 ± 3/12.59 ± 2.92(h)	Ischemic	MA/UT	SJ5, BL62, HT1, LU5, SP6, SP10	5	30	4
Liu et al. (103)	China	prospective	51/51	65.17 ± 11.82/64.25 ± 13.12	44.67 ± 1.52/4.48 ± 1.26	Ischemic	MA/UT	GV24, GV20, GV26, GV29, LI11, PC6, LI10, HT7, LI4, ST36, SP6, KI3, LR3	7	30	2
Lin et al. (100)	China	prospective	51/51	63.45 ± 3.88/63.54 ± 3.91	50.58 ± 5.16/50.62 ± 5.17	Mixed	MA/UT	GV24, GV26, GB20, GV20, BL7, GV14, BL10, BL8, BL17, BL15, BL18, BL20, CV4, BL23, BL54, SI12, SI9	5	30	4
Lin et al. (101)	China	prospective	50/50	50 ± 4/51 ± 4	33.5 ± 4.6/32.8 ± 6.7	Mixed	MA/UT	GV20, GV26, PC6, HT1, ST36, LI15, LI4, GB30, GB34	7	-	4
Liu et al. (102)	China	prospective	115/115	59.59 ± 7.2/60.23 ± 8.14	18.77 ± 4.87/18.98 ± 4.76	Mixed	MA/UT	EX-B2	6	30	2
Liu et al. (103)	China	prospective	50/50	64.12 ± 6.97/64.53 ± 6.32	16.93 ± 2.85/16.89 ± 3.47(weeks)	Mixed	MA/UT	GV24, GV29, GV16, GV20, GV26	7	30	4
Liu and Yu (104)	China	prospective	70/70	64.4 ± 6.6/65.2 ± 7.6	-	Ischemic	MA/UT	GV20, DU21, DU19, GV29, GV14, BL15, BL20, BL23	5	30	12
Liu et al. (105)	China	prospective	50/50	58.66 ± 4.19/59.02 ± 4.22	-	Hemorrhagic	MA/UT	PC6, GV26, SP6, HT1, LU5, BL40	7	-	4
Liu. (106)	China	prospective	60/60	60 ± 7/59 ± 8	19.63 ± 4.28/20.83 ± 4.72	Mixed	MA/UT	GV26, GV20, GB20, EX-HN1, GV29, GV24, LI4, LI15, SJ5, LI14, LI10, PC2, LI11, LU5, ST41, ST34, GB39, GB34, ST40, SP9, SP6, BL57, ST36	5	20	4
Liu et al. (107)	China	prospective	80/80	56.85 ± 13.85/59.63 ± 14.52	-	Ischemic	EA/UT	SJ10, SJ5, GB34	6	30	4
Liu et al. (108)	China	prospective	80/80	60.23 ± 6.37/59.77 ± 6.54	3.12 ± 3.63/2.84 ± 3.86(momths)	Mixed	MA/UT	HT1, LU5, PC7, PC6, BL37, ST32, GB34, ST41, BL62, LI15, LI11, LI10, SJ5, LI4, SI3, BL40, BL57, SP10, SP9, SP6, KI6	6	20	4
Liu et al. (109)	China	prospective	62/62	68.37 ± 7.97/68.37 ± 7.97	5.93 ± 1.58/5.76 ± 1.72(momths)	Ischemic	MA/UT	PC6, HT1, LU5, PC7, LI4, GB34, ST41, BL37	3	30	13
Liu et al. (110)	China	prospective	60/60	65.8 ± 5.67/65.94 ± 5.71	57.2 ± 8.37/58.79 ± 8.45(h)	Ischemic	EA/UT	The area related to the head acupoints	6	30	2
Liu et al. (47)	China	prospective	58/58	58.51 ± 8.90/58.46 ± 8.86	15.28 ± 3.69/15.31 ± 3.72(weeks)	Mixed	MA/UT	GV20, EX-HN1, GV24, GV17, HT7, LI4, SP6, GB39, KI3, LR3	6	30	4
Liu et al. (111)	China	prospective	60/60	57.83 ± 5.64/58.42 ± 6.33	6.04 ± 2.15/6.13 ± 1.87	Ischemic	WNM/UT	LI10, LI11, SJ5, LI15, LI4, ST36, BL60, ST34, GB34	5	40	12
Liu and Feng (160)	China	prospective	59/59	68.02 ± 5.73/66.18 ± 6.04	7.55 ± 1.89/7.09 ± 2.16	Mixed	MA/UT	HT7, ST40, LR3, BL58, LI4, SP3, GB20, KI3, GV24, EX-HN1, GV20, GV29	6	40	4
Liu et al. (112)	China	prospective	120/120	65.2 ± 5.4/67.4 ± 5.1	3.5 ± 0.8/3.4 ± 0.7	Ischemic	MA/UT	GV26, PC6, SP6, HT1, LU5, BL40, LI4	7	20	3
Lu et al. (113)	China	prospective	55/55	51.65 ± 9.36/53.56 ± 10.65	1182.96 ± 24.15/186.26 ± 27.65	Mixed	FN/MA	LI15, LI14, LI11, LI10, LI4, SJ5, SI3, SJ4, BL40, GB34, ST36, KI17, SP10, SP6, KI3	6	5	4
Luan and Xu (114)	China	prospective	72/72	56.89 ± 10.12/57.63 ± 9.45	4.19 ± 1.08/4.21 ± 1.23	Ischemic	PEA/UT	Eye area acupoints	7	-	2
Luo et al. (161)	China	prospective	94/94	71 ± 7/70 ± 7	66.81 ± 31.89/67.71 ± 33.10	Mixed	EA/UT	GB20, GV16, GV14	5	30	8
Ma et al. (115)	China	prospective	54/54	69.83 ± 5.63/68.78 ± 5.28	2.25 ± 0.52/2.35 ± 0.63(momths)	Ischemic	MA/UT	The area related to the head acupoints	5	30	8
Meng and Liu (116)	China	prospective	60/60	58 ± 15.39/57.35 ± 13.96	3.34 ± 1.77/3.54 ± 1.72(momths)	Mixed	PEA/MA	Eye area acupoints	5	30	3
Mu et al. (28)	China	prospective	67/68	59.4 ± 11.26/59.2 ± 10.38	-	Ischemic	MA/UT	PC6, GV26, GV20, EX-HN1, GB20, LI4, SP6, LR3	7	30	5
Qin et al. (117)	China	prospective	51/51	67.86 ± 3.54/68.37 ± 4.29	5.42 ± 1.45/5.63 ± 1.72(momths)	Ischemic	EA/UT	SP9, LI4, BL60, LI11, SJ10, SJ5, PC6	-	30	8
Ruan et al. (118)	China	prospective	50/50	68.99 ± 2.71/69.73 ± 2.65	2.31 ± 0.49/2.22 ± 0.45(momths)	Mixed	MA/UT	GV20, LI10, LI11, SJ5, LI4, ST36, SP6, LR3, GB34	5	30	3
Rui et al. (119)	China	prospective	180/180	54.15 ± 1.54/56.73 ± 3.21	1.02 ± 0.57/0.89 ± 0.17(momths)	Mixed	WNM/UT	GV29, GV20, EX-HN1, TE14, LI15, LI13, SI9, LI11, LI10, SJ5, LI4, EX-LE4, SP10, GB34, ST36, SP6, ST34, KI3, ST35, LR3, CV4, CV6	7	10	4
Sun et al. (120)	China	prospective	51/51	57.81 ± 4.29/57.86 ± 4.25	17.32 ± 2.65/17.37 ± 2.64(h)	Ischemic	WNM/UT	EX-HN1, LI15, LI14, LI10, SJ6, LI4, GB31, ST34, SP10, GB34, SP9, SP6, LR3	4	30	4
Sun et al. (121)	China	prospective	51/51	57.67 ± 9.21/57.67 ± 9.21	-	Ischemic	SA/UT	ST40, ST36, SP6, CV12	7	40	4
Sun et al. (122)	China	prospective	56/56	57.51 ± 9.09/58.22 ± 8.78	3.28 ± 0.85/3.03 ± 0.93(momths)	Ischemic	EA/UT	Eye area acupoints	7	45	4
Zhan et al. (23)	China	prospective	54/54	56.3 ± 4.8/57.2 ± 5.1	-	Ischemic	SA/UT	MS6, GV20, GB6	5	30	16
Zhan et al. (22)	China	prospective	51/51	60 ± 8/59 ± 8	-	Ischemic	MA/UT	GV26, CV12, CV6, PC6, ST25, LI4, ST36, GB34, SP6, LR3	7	30	2
Song et al. (19)	China	prospective	71/70	63 ± 6/64 ± 77	-	Ischemic	MA/UT	GV26, PC6, SP6, HT1, LU5, BL40	6	30	1
Li et al. (18)	China	prospective	159/165	64.3 ± 8.3/65.4 ± 6.9	3.49 ± 3.11/3.83 ± 3.25(momths)	Ischemic	MA/UT	GV20, GV26, PC9, ST6, ST4, LI15, LI11, LI4, GB30, GB31, GB34, GB39	5	30	2
Wang et al. (20)	China	prospective	60/60	62.4 ± 9/59.5 ± 8.9	3.3 ± 1.6/3.0 ± 1.56	Ischemic	SA/UT	MS6	6	30	2
Wang et al. (26)	China	prospective	60/60	62 ± 10/63 ± 8	2.7 ± 1.6/2.3 ± 1.5(momths)	Mixed	SA/UT	The area related to the head acupoints	7	60	8
Chen et al. (123)	China	prospective	120/124	62.52 ± 10.6/64.06 ± 10.54	4.53 ± 1.13/4.48 ± 1.32	Ischemic	MA/UT	MS6, MS7, LI15, LI11, LI10, SJ5, LI4, ST34, ST36, GB34, SP6, GB20, EX-HN14, BL10, GV16, CV23, GV20, GV24, GB13, EX-HN1	7	30	7
Teng et al. (124)	China	prospective	55/55	59.47 ± 8.62/58.65 ± 8.17	60.17 ± 10.68/59.67 ± 11.28	Mixed	PEA/UT	Eye area acupoints	7	5	4
Tian et al. (125)	China	prospective	98/98	62.81 ± 1.67/62.49 ± 1.73	-	Ischemic	MA/UT	EX-HN1, GV20, GV26, GB20, EX-HN5, LI15, SJ5, LI4, HT5, GB30, ST36, LI10, GB34, SP6,	7	20	4
Wang et al. (21)	China	prospective	79/79	60.75 ± 3.82/61.23 ± 3.65	-	Hemorrhagic	MA/UT	LI15, LI10, LI11, LI4, SJ23, SJ5, SI3, LI14, GB30, ST36, GB34, ST41, BL60, GB31, LR3	7	30	2
Wang et al. (27)	China	prospective	60/60	61.23 ± 2.03/61.27 ± 2.33	-	Hemorrhagic	MA/UT	GV26, PC6, BL40, LU5, HT1	7	30	12
Wang et al. (126)	China	prospective	65/65	72.09 ± 3.43/71.40 ± 3.48	3.15 ± 0.78/2.98 ± 0.67(momths)	Ischemic	PEA/UT	Eye area acupoints	5	30	4
Wang and Li (127)	China	prospective	64/64	71.42 ± 8.67/69.33 ± 7.56	67.57 ± 19.42/63.34 ± 17.37	Ischemic	SA/UT	GV26, PC6, SP6, GV20, DU21	5	40	10
Wang et al. (128)	China	prospective	120/120	-	-	Mixed	PEA/UT	Eye area acupoints	7	45	4
Wang et al. (129)	China	prospective	59/59	68.88 ± 3.64/67.71 ± 3.02	2.09 ± 0.63/2.30 ± 0.65	Ischemic	MA/UT	GV20, GB20, GV24, GV16, GV14, EX-B2, LI11, PC6, GB31, ST36, GB34, SP6, SP10	7	30	4
Wang et al. (130)	China	prospective	70/70	61.21 ± 5.43/60.95 ± 5.82	2.01 ± 0.5/2.13 ± 0.68(momths)	Mixed	MA/UT	LI4, LI15, SJ5, LI11, LI10, GB30, ST36, GB34, LR3, GB39, SP6	7	30	4
Wang et al. (131)	China	prospective	50/50	53.8 ± 11.7/54.5 ± 13.6	-	Mixed	SA/UT	The area related to the head acupoints	6	180	3
Wang et al. (29)	China	prospective	51/50	64.02 ± 8.01/62.47 ± 7.28	6.16 ± 1.22/6.25 ± 1.17	Mixed	MA/UT	PC6, GV26, SP6, LU5, HT1, BL40	5	30	6
Wang et al. (132)	China	prospective	56/54	65.8 ± 10.4/64 ± 10.6	45.9 ± 5.6/48.5 ± 9.6	Ischemic	FN/MA	PC6, SP6, HT1, LU5, BL40, LI15, SJ5, GB30, ST41, BL60	5	30	12
Wang et al. (133)	China	prospective	90/90	-	-	Mixed	EA/UT	LI15, LI13, LI11, LI10, SJ5, LI4, SP10, GB34, SP9, ST36, ST40, SP6, LR3	7	15	12
Wang et al. (134)	China	prospective	60/60	58.78 ± 9.68/60.48 ± 11.44	2.55 ± 1.47/2.83 ± 1.65(years)	Ischemic	PEA/UT	Eye area acupoints	5	30	2
Wang et al. (135)	China	prospective	65/65	60.5 ± 12.6/61.7 ± 10.9	3.3 ± 1.8/3.1 ± 1.5	Mixed	MA/UT	GV20, LI15, LI11, LI4, LI14, LI10, SJ5, ST34, ST36, ST40, GB39, ST41, GB40	7	30	4
Wei and Li (136)	China	prospective	100/100	63.64 ± 10.33/60.18 ± 9.46	17,224 ± 5.93/16.75 ± 5.61	Ischemic	MA/UT	GB20, EX-B2	6	30	2
Wei and Su (137)	China	prospective	50/50	51.41 ± 9.88/51.47 ± 10.05	6.77 ± 1.38/6.84 ± 1.47(momths)	Mixed	MA/UT	LI14, SJ5, LI15, LI4, LI11, LI10, GB31, ST36, GB34, LR3, SP10, SP6	3	30	4
Wu et al. (138)	China	prospective	60/60	44.49 ± 2.29/44.52 ± 2.23	3.24 ± 0.42/3.33 ± 0.37	Mixed	MA/UT	LI15, LI11, LI10, SJ6, SP9, ST36, SP6, KI3	7	30	2
Xiao. (139)	China	prospective	54/54	-	-	Ischemic	SA/UT	The area related to the head acupoints	5	60	12
Xiao et al. (140)	China	prospective	50/50	60.36 ± 8.79/59.84 ± 8.65	36.62 ± 11.68/34.84 ± 10.68	Mixed	EA/UT	PC6, SP6, EX-HN1, KI1, HT1	6	30	4
Xu et al. (141)	China	prospective	60/60	65.07 ± 7.1/64.78 ± 9.63	3.07 ± 2.73/3.21 ± 2.61	Ischemic	MA/UT	GV20, SP6, KI3, SP10, ST40, ST36	3	30	24
Xu et al. (142)	China	prospective	50/50	62.8 ± 4.1/63.2 ± 3.5	4.6 ± 0.8/4.8 ± 1.1(momths)	Ischemic	MA/UT	PC6, SP6, GV26, GB20, HT1, LU5, BL40, BL10	5	20	24
Xu et al. (143)	China	prospective	50/50	60.22 ± 9.14/59.24 ± 9.73	23.14 ± 7.82/24.65 ± 8.52	Ischemic	MA/UT	GV29, GV23, PC6, SP6, HT1, LU5, BL40, LI10, BL36	7	30	4
Yang et al. (144)	China	prospective	64/64	65.78 ± 12.35/66.12 ± 11.45	-	Hemorrhagic	SA/UT	GV20, EX-HN5	6	30	4
Yang et al. (145)	China	prospective	50/50	63.52 ± 1.47/63.91 ± 1.55	2.53 ± 0.61/2.7 ± 0.42(h)	Ischemic	MA/UT	GV20, GB20, PC6, SP6, GV26, BL40, LU5, HT1	7	30	2
Zhang et al. (146)	China	prospective	58/62	56.6 ± 2.35/56.52 ± 2.31	3.12 ± 0.45/3.38 ± 0.53(momths)	Mixed	MA/UT	LI4, PC6, LI11, LI10, LU5, LR3, GB40, GB34, SP10, GB30	7	20	4
Zhang et al. (147)	China	prospective	50/50	64.5 ± 7.42/65.23 ± 10.75	15.39 ± 2.73/16.21 ± 2.12(years)	Ischemic	MA/UT	PC6, GV26, SP6, HT1, LU5, BL40, ST36	7	30	4
Zhang et al. (148)	China	prospective	50/50	56.38 ± 5.67/56.24 ± 5.82	-	Hemorrhagic	MA/UT	SP9, ST36, CV12, ST40, SP3, GB43, LR3, GB20, LU5, HT1, BL40	7	30	24
Zhang. (149)	China	prospective	65/65	54.18 ± 3.51/53.94 ± 3.33	20.62 ± 11.91/19.83 ± 12.07	Mixed	MA/UT	GV20, GB20	7	30	6
Zhang et al. (4)	China	prospective	60/60	63.8 ± 10/61.9 ± 12.3	2.57 ± 0.81/2.37 ± 0.58(momths)	Mixed	SA/UT	GV20, DU21, GV24, EX-HN5, GV29, GV26, ST2, GV14, GB20, ST40	5	30	3
Zhang and Tian (150)	China	prospective	50/50	56 ± 12/55 ± 13	2.68 ± 0.61/2.73 ± 0.64(h)	Ischemic	MA/UT	GV26, PC6, SP6, HT1, BL40, LU5	7	-	2
Zhao et al. (151)	China	prospective	50/50	60.88 ± 17.09/58.39 ± 15.22	20.78 ± 6.36/19.20 ± 6.12	Ischemic	PEA/UT	Eye area acupoints	7	30	2
Zhen and Zhang (152)	China	prospective	50/50	59.23 ± 7.54/60.13 ± 7.21	25.43 ± 9.42/26.68 ± 10.34(h)	Ischemic	MA/UT	GV26, SP6, PC6, GB20, HT1, LU5, BL40	6	20	2
Zhen et al. (153)	China	prospective	56/60	60.98 ± 5.85/60.85 ± 5.74	5.41 ± 0.99/5.23 ± 0.98(momths)	Mixed	MA/UT	The area related to the head acupoints	5	30	4
Zhou et al. (154)	China	prospective	60/60	61.44 ± 8.77/62.04 ± 8.69	5.42 ± 1.87/5.37 ± 1.98(momths)	Mixed	EA/UT	GV20, GV24	6	30	4
Zhou et al. (24)	China	prospective	51/52	-	-	Ischemic	EA/UT	GV20, EX-HN1, PC6, SP6, ST36, GB34, LI10, LI11	7	30	4
Zhou et al. (155)	China	prospective	50/50	60.72 ± 6.99/59.60 ± 6.89	2.46 ± 0.84/2.70 ± 0.81(momths)	Mixed	SA/UT	The area related to the head acupoints	6	30	4
Zhou et al. (156)	China	prospective	80/80	55.25 ± 9.39/54.73 ± 10.42	-	Ischemic	WNM/UT	GV20, EX-HN1, LI15, TE14, LI11, LI10, PC6, LI4, GB30, GB34, ST36, SP6, BL60	7	30	8
Zhu. (157)	China	prospective	55/54	60.05 ± 8.67/59.58 ± 9.01	3.51 ± 0.89/3.73 ± 0.67(momths)	Ischemic	MA/UT	HT1, SP6, PC6, ST36, LI4, ST40	5	30	4
Zhu and Zhang (158)	China	prospective	61/65	55.83 ± 12.12/56.34 ± 11.72	28.72 ± 6.21/28.43 ± 7.32	Ischemic	SA/UT	The area related to the head acupoints	7	30	12
Zhu and Zhou (159)	China	prospective	60/60	62.1 ± 11.44/59.88 ± 11.54	29.70 ± 8.6/31.6 ± 8.79	Mixed	SA/UT	The area related to the head acupoints	5	30	4

MA, manual acupuncture; SA, scalp acupuncture; EA, electroacupuncture; WNM, warming needle moxibustion; PEA, peri-eye acupuncture; FN, fire needling.

### 3.2 Quality assessment of included studies

**Figure 2:**
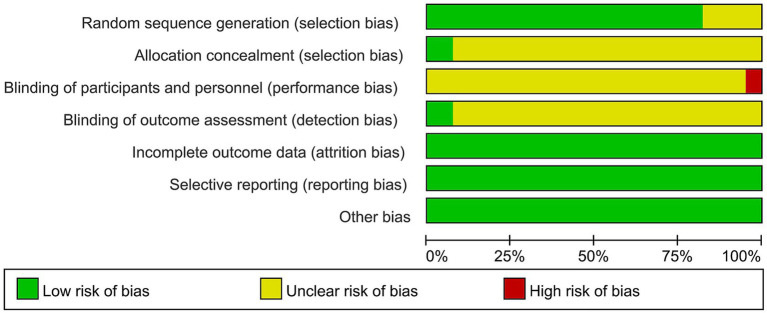
The risk diagram of bias included in the study.

**Figure 3:**
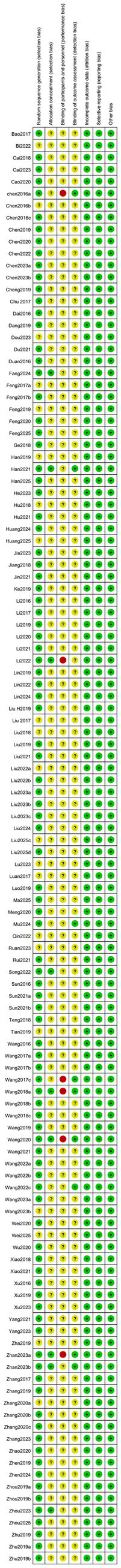
Summary chart of bias risks included in the study.

The methodological assessment revealed that 99 trials documented explicit randomization protocols, principally utilizing random number tables, warranting low-risk classification. Studies referencing only “randomized” without methodological details were designated as unclear risk. Appropriate allocation concealment through opaque sealed envelopes was implemented in nine studies (16–24), meriting low-risk designation, whereas insufficiently documented trials were classified as unclear. Pertaining to blinding implementation, six studies (18, 20, 23, 25–27) recognized the inherent limitations of blinding in acupuncture trials and were accordingly assigned high-risk status. Nine studies (17, 20–23, 25, 26, 28, 29) maintained blinded endpoint evaluation, achieving low-risk status, while inadequately described studies were categorized as unclear. Comprehensive outcome reporting without selective bias was consistently observed across all studies, resulting in uniform low-risk ratings for these domains (see Figures 2, 3).

### 3.3 Characteristics of included studies

**Figure 4:**
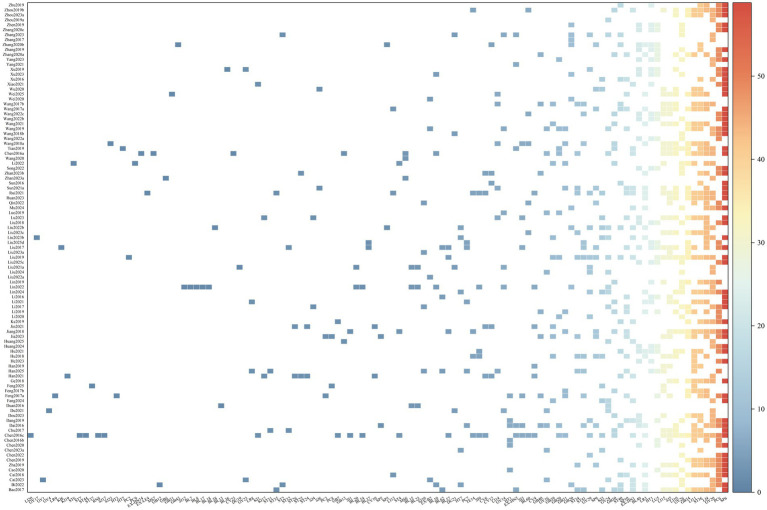
Acupoints heatmap.

**Figure 5:**
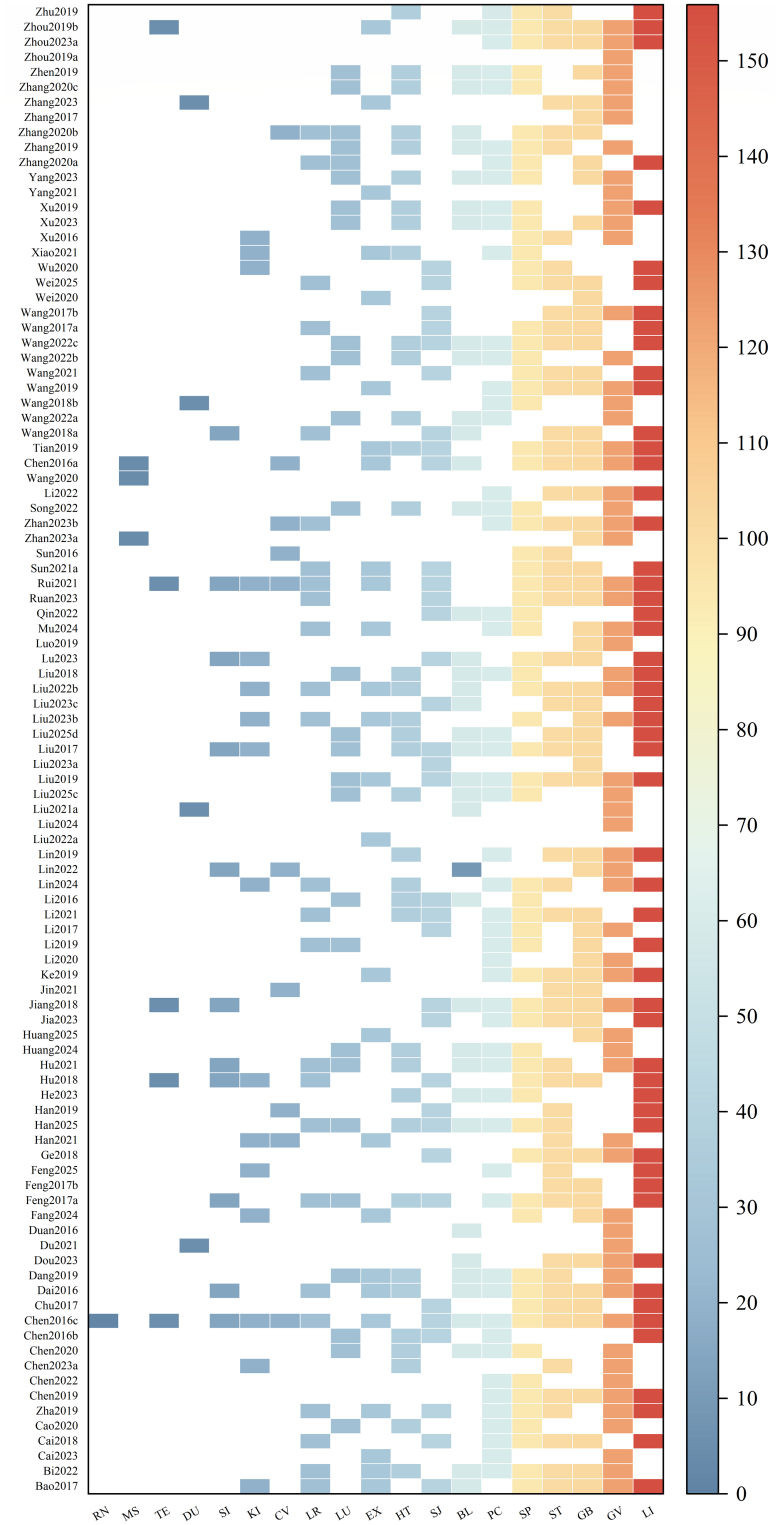
Meridian heatmap.

The final analysis encompassed 120 randomized controlled trials, with a collective enrollment of 15,848 stroke patients. The distribution of participants resulted in 7,911 individuals in the intervention groups and 7,937 in control conditions. The control groups encompassed six treatment modalities: MA, SA, EA, WNM, PEA, and FN. A heat map was generated to illustrate the utilization patterns of acupoints and meridians (see Figures 4, 5). The analysis identified a total of 144 distinct acupoints and 19 meridians. Among these, Sanyinjiao (SP6) and Neiguan (PC6) were the most frequently selected acupoints, whereas the Large Intestine Meridian (LI) and the Governor Vessel (GV) constituted the predominant meridians in the included protocols.

### 3.4 Network meta-analysis

#### 3.4.1 National institutes of health stroke scale

**Figure 6:**
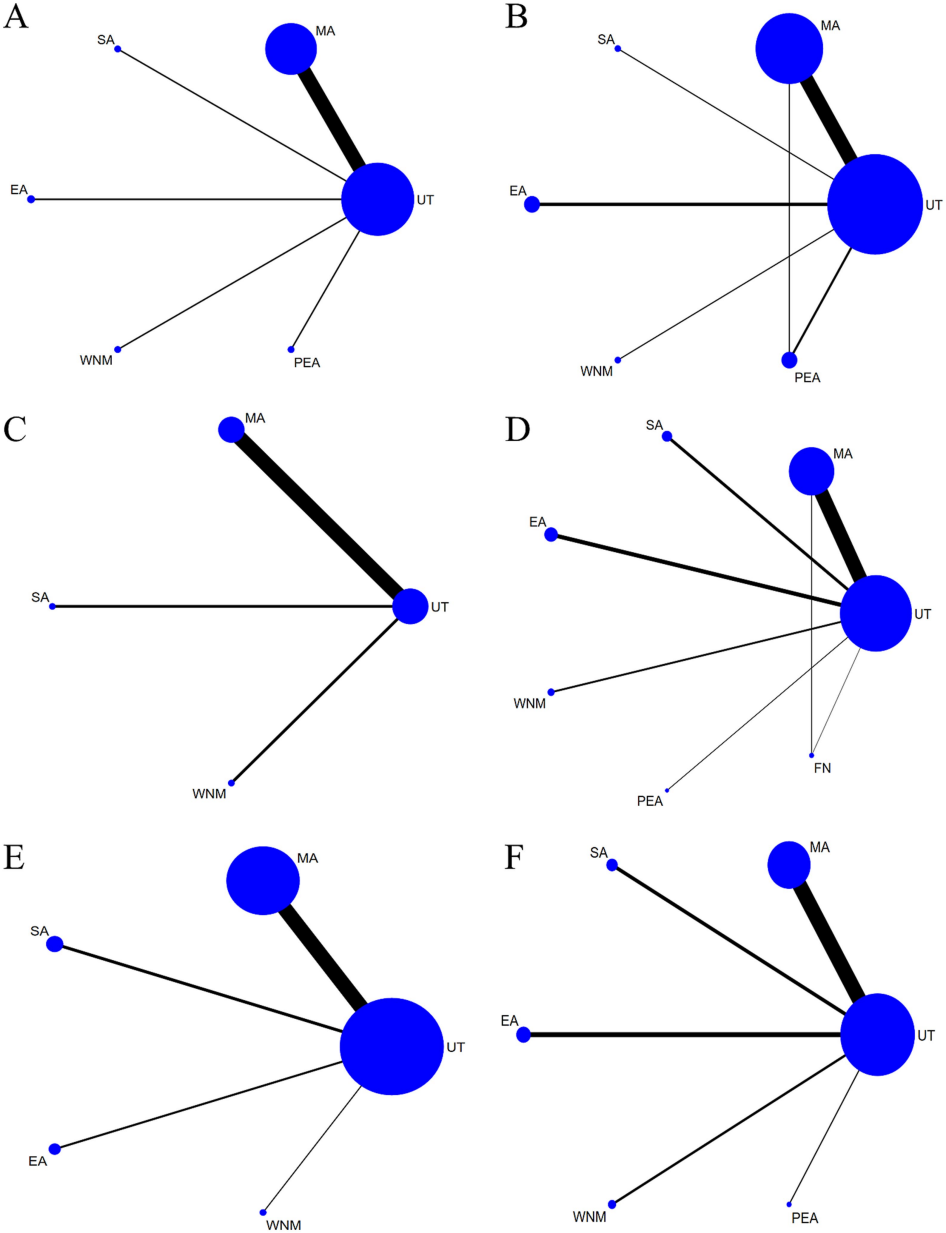
**(A)** NMA figure for NIHSS. **(B)** NMA figure for mRS. **(C)** NMA figure for ADLs. **(D)** NMA figure for Barthel Index. **(E)** NMA figure for MOCA. **(F)** NMA figure for MMSE.

Sixty-eight studies reported NIHSS as an outcome measure. The network evidence diagram (see Figure 6) involved five acupuncture interventions: MA, SA, EA, WNM, and PEA. The absence of closed loops within the network structure allowed for the implementation of a consistency model in the analytical approach.

In the network meta-analysis, three interventions—MA (SMD = −0.51, 95% CI: −0.74 to −0.28), EA (SMD = −1.48, 95% CI: −2.25 to −0.72), and WNM (SMD = −1.00, 95% CI: −1.71 to −0.30)—yielded favorable results against the control group for NIHSS improvement. Additionally, EA exhibited statistically significant advantages over SA (SMD = −1.09, 95% CI: −2.13 to −0.05), MA (SMD = −0.98, 95% CI: −1.77 to −0.18), and PEA (SMD = −1.26, 95% CI: −2.30 to −0.22; *p* < 0.05). Other comparative analyses showed non-significant results (*p* > 0.05; see Table 3).

**Table 3:** Network meta-analysis of NIHSS and ADLs scores.

UT	MA	SA	EA	WNM	PEA
**UT**	**0.87 (0.62,1.11)** ^*^	0.61 (−0.31,1.53)	**0.90 (0.38,1.42)** ^*^	**1.11 (0.20,2.02)** ^*^	**0.53 (0.01,1.05)** ^*^
**0.51 (0.28,0.74)** ^*^	**MA**	−0.26 (−1.21,0.69)	0.03 (−0.54,0.61)	0.24 (−0.70,1.18)	−0.34 (−0.87,0.20)
0.40 (−0.31,1.10)	−0.11 (−0.86,0.63)	**SA**	0.29 (−0.77,1.35)	0.50 (−0.79,1.79)	−0.08 (−1.13,0.97)
**1.48 (0.72,2.25)** ^*^	**0.98 (0.18,1.77)** ^*^	**1.09 (0.05,2.13)** ^*^	**EA**	0.21 (−0.84,1.26)	−0.37 (−1.11,0.37)
**1.00 (0.30,1.71)** ^*^	0.49 (−0.25,1.24)	0.61 (−0.39,1.61)	−0.48 (−1.52,0.56)	**WNM**	−0.58 (−1.63,0.46)
0.22 (−0.48,0.93)	−0.29 (−1.03,0.45)	−0.17 (−1.17,0.83)	**−1.26 (−2.30,−0.22)** ^*^	−0.78 (−1.77,0.22)	**PEA**

The blue and pink areas indicate improvement in NIHSS (lower left) and ADLs (upper right). Bold values and *indicate statistical significance (*P* < 0.05).

**Figure 7:**
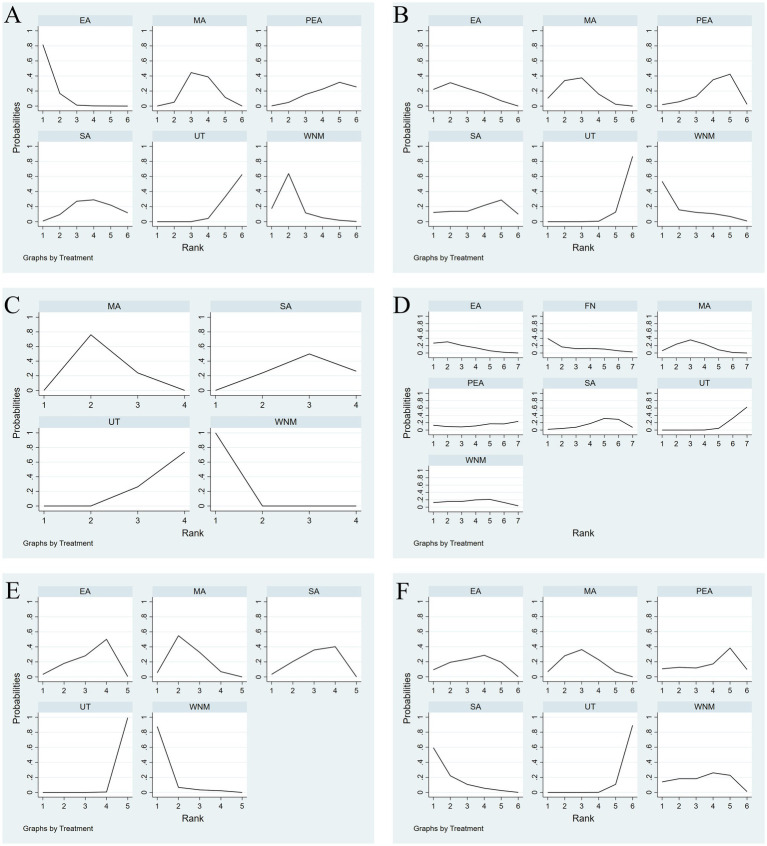
**(A)** SUCRA plot for NIHSS. **(B)** SUCRA plot for mRS. **(C)** SUCRA plot for ADLs. **(D)** SUCRA plot for Barthel index. **(E)** SUCRA plot for MOCA. **(F)** SUCRA plot for MMSE.

The SUCRA probability ranking for different acupuncture methods in reducing NIHSS in stroke patients was as follows: EA (95.7%) > WNM (78%) > MA (50.1%) > SA (40%) > PEA (28.1%) > UT (8.1%), see Figure 7.

#### 3.4.2 Activities of daily living scale

Twenty-one studies reported ADL. The network evidence diagram (see Figure 6) involved five acupuncture therapies: MA, SA, EA, WNM, and PEA, forming one triangular closed loop. Global and local inconsistency tests indicated good consistency (*p* > 0.05), thus a consistency model was used for analysis.

In the network meta-analysis, four acupuncture modalities—MA (SMD = 0.87, 95% CI: 0.62 to 1.11), EA (SMD = 0.9, 95% CI: 0.38 to 1.42), WNM (SMD = 1.11, 95% CI: 0.2 to 2.02), and PEA (SMD = 0.53, 95% CI: 0.01 to 1.05)—all demonstrated statistically significant advantages over conventional treatment in improving ADL scores (*p* < 0.05). The remaining interventional comparisons did not reach statistical significance (*p* > 0.05; see Table 3).

The SUCRA probability ranking for different acupuncture methods in improving ADL in stroke patients was as follows: WNM (80.1%) > EA (68%) > MA (67.1%) > SA (45.6%) > PEA (36.6%) > UT (2.5%), see Figure 7.

#### 3.4.3 Modified Rankin scale

Six studies provided mRS outcomes. The network evidence structure incorporated three acupuncture interventions—MA, SA, and EA—without forming closed loops, thus supporting the application of a consistency model for analysis (see Figure 6).

The network meta-analysis revealed that both MA (SMD = −0.30, 95% CI: −0.48 to −0.11) and WNM (SMD = −1.44, 95% CI: −1.67 to −1.2) demonstrated significant advantages over conventional treatment in reducing mRS scores. Furthermore, WNM showed statistically superior efficacy compared to both MA (SMD = −1.14, 95% CI: −1.44 to −0.84) and SA (SMD = −1.31, 95% CI: −1.75 to −0.87), with all reported differences reaching statistical significance (*p* < 0.05). The remaining comparative analyses yielded non-significant results (*p* > 0.05; see Table 4).

**Table 4:** Network meta-analysis of mRS and MOCA scores.

UT	MA	SA	EA	WNM
**UT**	**0.57 (0.44,0.70)** ^*^	**0.47 (0.18,0.76)** ^*^	**0.44 (0.11,0.77)** ^*^	**0.95 (0.43,1.47)** ^*^
**0.30 (0.11,0.48)** ^*^	**MA**	−0.11 (−0.42,0.21)	−0.13 (−0.49,0.23)	0.38 (−0.16,0.92)
0.13 (−0.25,0.50)	−0.17 (−0.59,0.25)	**SA**	−0.03 (−0.47,0.42)	0.48 (−0.12,1.08)
**-**	-	-	**EA**	0.51 (−0.11,1.13)
**1.44 (1.20,1.67)** ^*^	**1.14 (0.84,1.44)** ^*^	**1.31 (0.87,1.75)** ^*^	-	**WNM**

The blue and pink areas indicate improvement in mRS (lower left) and MOCA (upper right). “-” indicates no relevant comparison. Bold values and *indicate statistical significance (*P* < 0.05).

The SUCRA probability ranking for different acupuncture methods in reducing mRS scores in stroke patients was as follows: WNM (100%) > MA (59.8%) > SA (31.4%) > UT (8.8%), see Figure 7.

#### 3.4.4 Barthel index

Sixty-seven studies reported the BI. The network evidence diagram (see Figure 6) involved six acupuncture therapies: MA, SA, EA, WNM, PEA, and FN, forming one triangular closed loop. Global and local inconsistency tests indicated good consistency (*p* > 0.05), thus a consistency model was used for analysis.

According to the network meta-analysis, two acupuncture modalities demonstrated statistically significant improvements in the Barthel Index compared to conventional treatment: MA (SMD = 0.80, 95% CI: 0.52 to 1.07) and EA (SMD = 0.91, 95% CI: 0.40 to 1.43). No other interventional comparisons reached the threshold for statistical significance (*p* > 0.05; see Table 5).

**Table 5:** Network meta-analysis of BI index and MMSE scores.

UT	MA	SA	EA	WNM	PEA	FN
**UT**	**0.54 (0.38,0.70)** ^*^	**0.72 (0.38,1.05)** ^*^	**0.49 (0.20,0.79)** ^*^	**0.49 (0.09,0.90)** ^*^	0.38 (−0.19,0.96)	-
**−0.80 (−1.07,-0.52)** ^*^	**MA**	−0.38 (−1.04,0.27)	0.13 (−0.45,0.71)	−0.12 (−0.93,0.68)	−0.34 (−1.57,0.89)	-
−0.40 (−1.00,0.21)	0.04 (−0.27,1.06)	**SA**	0.52 (−0.28,1.31)	0.26 (−0.71,1.23)	0.05 (−1.29,1.39)	-
**−0.91 (−1.43,-0.40)** ^*^	−0.12 (−0.70,0.47)	−0.52 (−1.31,0.28)	**EA**	−0.26 (−1.17,0.66)	−0.47 (−1.77,0.84)	-
−0.66 (−1.42,0.11)	0.14 (−0.68,0.95)	−0.26 (−1.24,0.72)	0.26 (−0.67,1.18)	**WNM**	−0.21 (−1.63,1.21)	-
−0.45 (−1.66,0.76)	0.35 (−0.89,1.59)	−0.05 (−1.40,1.30)	0.47 (−0.85,1.78)	0.21 (−1.22,1.64)	**PEA**	-
−0.93 (−1.94,0.07)	−0.13 (−1.13,0.86)	−0.53 (−1.71,0.64)	−0.02 (−1.15,1.11)	−0.27 (−1.54,0.99)	−0.48 (−2.06,1.09)	**FN**

The blue and pink areas indicate improvement in BI index (lower left) and MMSE (upper right). “-” indicates no relevant comparison. Bold values and *indicate statistical significance (*P* < 0.05).

The SUCRA probability ranking for different acupuncture methods in improving the BI in stroke patients was as follows: EA (74.8%) > FN (70.7%) > MA (66.2%) > WNM (54.5%) > PEA (42.1%) > SA (35.1%) > UT (6.9%), see Figure 7.

#### 3.4.5 Montreal cognitive assessment

Twenty studies provided MoCA outcomes. The network evidence structure incorporated four acupuncture interventions—MA, SA, EA, and WNM—without forming closed loops (see Figure 6), supporting the application of a consistency model for analysis.

The network meta-analysis demonstrated that all four evaluated acupuncture modalities showed statistically significant improvements in MoCA scores compared to conventional treatment: MA (SMD = 0.57, 95% CI: 0.44 to 0.7), SA (SMD = 0.47, 95% CI: 0.18 to 0.76), EA (SMD = 0.44, 95% CI: 0.11 to 0.77), and WNM (SMD = 0.95, 95% CI: 0.43 to 1.47). The remaining comparative analyses did not reach statistical significance (*p* > 0.05; see Table 4).

The SUCRA probability ranking for different acupuncture methods in improving MoCA scores in stroke patients was as follows: WNM (94.9%) > MA (64.7%) > SA (46.8%) > EA (43.4%) > UT (0.1%), see Figure 7.

#### 3.4.6 Mini-mental state examination

Twenty-three studies utilized the MMSE as an outcome measure. The network evidence structure incorporated five acupuncture interventions—MA, SA, EA, WNM, and PEA—without forming closed loops (see Figure 6), thus supporting the application of a consistency model for analysis.

The network meta-analysis revealed that four acupuncture modalities demonstrated statistically significant superiority over conventional treatment in improving MMSE scores: MA (SMD = 0.54, 95% CI: 0.38 to 0.7), SA (SMD = 0.72, 95% CI: 0.38 to 1.05), EA (SMD = 0.49, 95% CI: 0.2 to 0.79), and WNM (SMD = 0.49, 95% CI: 0.09 to 0.9). The remaining comparative analyses did not reach statistical significance (*p* > 0.05; see Table 5).

The SUCRA probability ranking for different acupuncture methods in improving MMSE scores in stroke patients was as follows: SA (86.1%) > MA (61.1%) > WNM (54.3%) > EA (54.1%) > PEA (42.3%) > UT (2.2%), see Figure 7.

### 3.5 Publication bias

**Figure 8:**
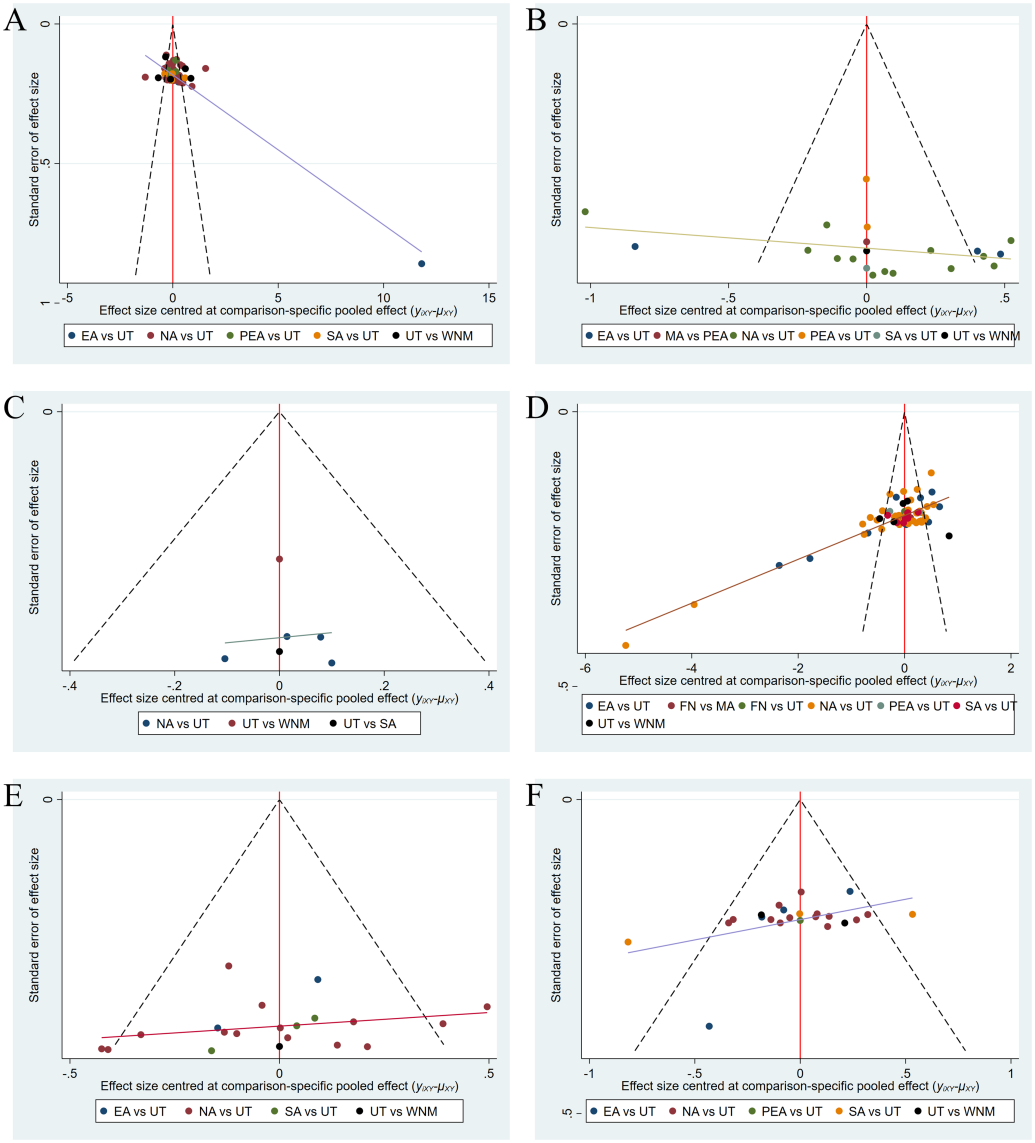
The funnel plots for publication bias of **(A)** NIHSS, **(B)** mRS, **(C)** ADLs, **(D)** Barthel Index, **(E)** MoCA, and **(F)** MMSE.

Funnel plots were generated for all individual outcome indicators to assess potential publication bias. The dots in the plots represent the included studies. Visual inspection revealed suboptimal symmetry in the funnel plots, with some study points distributed outside the 95% CI, suggesting possible publication bias and small-study effects, see Figure 8.

### 3.6 Sensitivity analysis

To further verify the reliability of the findings, a sensitivity analysis was conducted by excluding 21 studies with an “unclear risk” of bias in random sequence generation and 6 studies with a “high risk” of bias in blinding of participants and personnel. The results of the network meta-analysis for all outcome indicators showed no directional changes across the remaining 93 high-quality studies. Furthermore, the SUCRA-based rankings for each intervention remained highly consistent with the primary analysis. These findings demonstrate that the conclusions of this study are robust and were not significantly influenced by studies of lower methodological quality.

## 4 Discussion

This systematic review and network meta-analysis provides a comprehensive comparison of six acupuncture modalities in stroke rehabilitation. The analysis indicated consistent superiority of all ATs over UT, with clear domain-specific superiority: EA was most effective for NIHSS and BI outcomes; WNM proved optimal for mRS and MoCA measures; while SA showed peak efficacy for MMSE scores.

EA exhibited significant advantages in ameliorating NIHSS scores and the BI index. As a modern acupuncture modality characterized by the application of pulsed current following Deqi (needling sensation), EA is capable of generating sustained and quantifiable stimulation (30). The NIHSS reflects the severity of neurological deficits (31), whereas the BI assesses basic activities of daily living (32). Following the onset of stroke, the infarct core undergoes rapid neuronal death resulting from severe hypoperfusion (33); conversely, the ischemic penumbra—where blood flow is maintained at approximately 60% of baseline levels—remains temporarily viable yet functionally impaired, thereby constituting a critical therapeutic window for intervention (34). EA has been shown to significantly enhance synaptic plasticity and upregulate the expression of key proteins such as SYN and PSD-95 in ischemic regions, thereby facilitating the repair and reconstruction of neural connections (35). Animal experiments have confirmed that low-intensity electroacupuncture stimulation at acupoints such as Shousanli and Zusanli significantly improved neurological function scores, reduced cerebral infarct volume, and alleviated histopathological damage in rat models of middle cerebral artery occlusion and reperfusion (36). Furthermore, EA promotes the release of neurotrophic factors, including BDNF and NGF, which support neuronal survival and axonal growth, while concurrently inducing angiogenesis to improve local blood supply and metabolic support (37). These mechanisms are consistent with the observed efficacy of EA in restoring neurological function and motor-related activities of daily living.

WNM ranked highest in ameliorating mRS, ADL, and MoCA scores. The mRS assesses the degree of global disability (38), the ADL scale objectively quantifies the patient’s capacity to perform basic activities of daily living (39), and the MoCA serves to detect mild cognitive impairment (40). Although the immediate threat to life is resolved during the acute phase, a majority of stroke patients continue to exhibit varying degrees of residual limb motor dysfunction and cognitive decline. The persistence of these sequelae not only impairs patients’ daily living but may also exacerbate progressively with the course of the disease, resulting in a significant deterioration in quality of life (41). The efficacy of WNM derives from the synergistic effects of acupuncture and moxibustion. Acupuncture modulates central nervous system function via neural reflex pathways by stimulating nerve endings surrounding acupoints, thereby promoting neural repair and regeneration; concurrently, the thermal effects generated during moxibustion induce local vasodilation and improve microcirculation, providing essential nutritional support for neuronal recovery (42). Furthermore, various bioactive components contained in moxa leaves possess anti-inflammatory and anticoagulant properties, as well as the ability to inhibit platelet aggregation (43). Both moxibustion and moxa smoke are also postulated to exert multi-target regulatory functions on the nervous and immune systems (44). Research indicates that WNM alleviates inflammatory responses and edema in brain tissue by inhibiting the release of pro-inflammatory cytokines (45). Simultaneously, it promotes the recovery of neurological function by improving cerebrovascular vasomotor function and hemorheological properties, enhancing blood perfusion in ischemic regions, and optimizing the metabolic environment for neuronal cells. Zhen et al. (46) noted that the combination of WNM and rehabilitation training promotes systemic metabolism and axonal regeneration, thereby contributing to the amelioration of muscle atrophy and the reconstruction of normal reflex arcs. Moreover, a systematic review demonstrated (47) that heat-sensitive moxibustion combined with cognitive training represents one of the optimal acupuncture regimens for improving MoCA scores in stroke patients, further supporting the positive role of WNM in cognitive rehabilitation.

SA exhibited the optimal potential for intervention in ameliorating MMSE scores in stroke patients. The MMSE serves to effectively reflect the cognitive status of patients (48). The therapeutic efficacy of SA is exerted through the stimulation of specific acupoint areas on the scalp. On the one hand, it improves cerebral microcirculation and enhances blood oxygen supply in ischemic regions (49); on the other hand, it achieves sustained protection of the nervous system by modulating hemorheological indices and neuroelectrophysiological activity (50). When acupuncture stimulation is applied to areas corresponding to the frontal and temporal lobes—regions closely associated with cognitive function—it effectively activates cerebral cortical function, modulates neuroinflammatory responses, and facilitates the repair and regeneration of damaged neural tissue, thereby improving cognitive performance (51). Experimental studies (52) have confirmed that stimulation of scalp acupoints such as Shenting and Baihui significantly attenuated neuroinflammatory responses in rats with ischemia–reperfusion injury and effectively improved learning and memory capabilities in model animals by regulating the expression levels of autophagy-related proteins, including PI3K/AKT/Beclin-1. Clinical observations conducted by Li et al. (53) on 73 patients with post-stroke cognitive impairment indicated that, following 4 weeks of SA treatment, the P300 latency was significantly shortened, the amplitude was markedly increased, and MMSE scores were significantly elevated. These findings suggest that scalp acupuncture treatment effectively ameliorates neuroelectrophysiological indices and promotes the recovery of cognitive function.

The heat map analysis identified the acupoints SP6 and PC6, along with the meridians LI and GV, as the core elements of the acupuncture prescriptions. SP6 represents the intersection point of the three Yin meridians of the foot (Spleen, Liver, and Kidney); stimulation of SP6 can simultaneously regulate and tonify the three viscera, thereby correcting imbalances in Qi, blood, Yin, and Yang. Furthermore, stimulation of SP6 activates and innervates related neurons within the same segment, effectively modulating the balance between excitation and inhibition in the cerebral cortex, promoting neural functional remodeling, and ameliorating limb motor function (54). PC6 serves as the Luo-connecting point of the Pericardium Meridian of Hand-Jueyin and is one of the Eight Confluent Points, connecting to the Yin Wei Vessel; it functions to dredge Qi and blood and communicate between Yin and Yang. Stimulation of PC6 has been demonstrated to improve cerebral perfusion in the ischemic penumbra and activate the parasympathetic nervous system, thereby creating a favorable metabolic environment for neuronal survival (55). The predominance of GV and LI corresponds to the Traditional Chinese Medicine theories of “unblocking the Governor Vessel and awakening the brain” and “treating flaccidity by selecting Yangming alone.” GV is closely associated with the function of the central nervous system. GV acupuncture significantly improves neurological outcomes in stroke patients, a benefit attributed to its potential mechanisms in inhibiting neuronal apoptosis and facilitating central nervous system plasticity (56). LI belongs to Yangming, which is abundant in Qi and blood, and serves as the preferred meridian for treating flaccidity syndromes such as post-stroke limb dysfunction (57). Stimulation of acupoints on the Li has been confirmed to facilitate the reorganization of the motor cortex and enhance the functional connectivity of the sensorimotor network (58).

The results of the present study provide evidence-based support for the selection of precise and individualized acupuncture regimens during stroke rehabilitation. Clinicians may prioritize EA for neurological deficits, whereas WNM or SA are recommended for cognitive impairment. Although the present study demonstrated good overall statistical consistency, the potential clinical heterogeneity among the included studies must be acknowledged. Variations in stroke type and disease duration may constitute primary sources of heterogeneity, as the sensitivity of patients to acupuncture treatment may differ across stages. Furthermore, discrepancies regarding intervention details and routine protocols existed among the studies. Consequently, when interpreting these rankings, it is essential to carefully distinguish between statistical significance and clinical relevance.

## 5 Strengths and limitations

The strengths of the present study include a comprehensive search of eight databases, thereby minimizing retrieval bias. By incorporating 120 RCTs with over 15,000 patients, six therapies were simultaneously ranked across core outcomes. However, several limitations must be acknowledged. First, as all included RCTs were conducted in China, potential racial physiological differences and cultural variations in acupuncture acceptance may limit external validity in non-Asian populations. Second, the limited number of studies for certain outcomes (notably mRS) resulted in sparse evidence networks and insufficient closed loops, warranting cautious interpretation. Finally, the scarcity of direct head-to-head comparisons between acupuncture modalities limited the precision of relative efficacy assessments. Future high-quality, multi-center global RCTs are required to verify these findings.

## Data Availability

The original contributions presented in the study are included in the article/Supplementary material, further inquiries can be directed to the corresponding authors.
