# Supplementary material for: Effects of multiple acupuncture therapies on cognitive function and quality of life in stroke patients: a systematic review and network meta-analysis
Source: Front Neurol. 2026 Mar 18;17:1764104. doi: 10.3389/fneur.2026.1764104 (PMC13038561; doi:10.3389/fneur.2026.1764104)
Supplement: Supplementary file 1 [file Data_Sheet_1.PDF]

Suppl. Table 1. Search strategy on CNKI and Wangfang.

Search strategy on CNKI and Wangfang	
#1	Acupuncture OR Manual acupuncture OR Scalp acupuncture OR Electroacupuncture OR Auricular acupuncture OR warming needle moxibustion OR Peri-eye acupuncture OR Body acupuncture OR Laser acupuncture
#2	Stroke OR Cerebral infarction OR Cerebrovascular accident OR Cerebral hemorrhage
#3	Random OR Randomized controlled trial
#4	#1 AND #2 AND #3
